# Diagnostic Accuracy of Real-Time PCR Assays Targeting 16S rRNA and *lipl32* Genes for Human Leptospirosis in Thailand: A Case-Control Study

**DOI:** 10.1371/journal.pone.0016236

**Published:** 2011-01-24

**Authors:** Janjira Thaipadunpanit, Wirongrong Chierakul, Vanaporn Wuthiekanun, Direk Limmathurotsakul, Premjit Amornchai, Siriphan Boonslip, Lee D. Smythe, Roongrueng Limpaiboon, Alex R. Hoffmaster, Nicholas P. J. Day, Sharon J. Peacock

**Affiliations:** 1 Mahidol-Oxford Tropical Medicine Research Unit, Faculty of Tropical Medicine, Mahidol University, Bangkok, Thailand; 2 Medical Proteomics Unit, Office for Research and Development, Faculty of Medicine, Siriraj Hospital, Bangkok, Thailand; 3 Department of Clinical Tropical Medicine, Faculty of Tropical Medicine, Mahidol University, Bangkok, Thailand; 4 Department of Tropical Hygiene, Faculty of Tropical Medicine, Mahidol University, Bangkok, Thailand; 5 Department of Microbiology and Immunology, Faculty of Tropical Medicine, Mahidol University, Bangkok, Thailand; 6 Communicable Disease Unit, WHO/FAO/OIE Collaborating Centre for Reference & Research on Leptospirosis, Western Pacific Region, Queensland Health Forensic and Scientific Services, Brisbane, Australia; 7 Udon Thani Regional Hospital, Udon Thani, Thailand; 8 National Center for Emerging and Zoonotic Infectious Diseases, Centers for Diseases Control and Prevention, Atlanta, Georgia, United States of America; 9 Nuffield Department of Clinical Medicine, Center for Clinical Vaccinology and Tropical Medicine, University of Oxford, Churchill Hospital, Oxford, United Kingdom; 10 Department of Medicine, University of Cambridge, Addenbrooke's Hospital, Cambridge, United Kingdom; University of Hyderabad, India

## Abstract

**Background:**

Rapid PCR-based tests for the diagnosis of leptospirosis can provide information that contributes towards early patient management, but these have not been adopted in Thailand. Here, we compare the diagnostic sensitivity and specificity of two real-time PCR assays targeting *rrs* or *lipL32* for the diagnosis of leptospirosis in northeast Thailand.

**Methods/Principal Findings:**

A case-control study of 266 patients (133 cases of leptospirosis and 133 controls) was constructed to evaluate the diagnostic sensitivity and specificity (DSe & DSp) of both PCR assays. The median duration of illness prior to admission of cases was 4 days (IQR 2–5 days; range 1–12 days). DSe and DSp were determined using positive culture and/or microscopic agglutination test (MAT) as the gold standard. The DSe was higher for the *rrs* assay than the *lipL32* assay (56%, (95% CI 47–64%) versus 43%, (95% CI 34–52%), p<0.001). No cases were positive for the *lipL32* assay alone. There was borderline evidence to suggest that the DSp of the *rrs* assay was lower than the *lipL32* assay (90% (95% CI 83–94%) versus 93%, (95%CI 88–97%), p = 0.06). Nine controls gave positive reactions for both assays and 5 controls gave a positive reaction for the *rrs* assay alone. The DSe of the *rrs* and *lipL32* assays were high in the subgroup of 39 patients who were culture positive for *Leptospira* spp. (95% and 87%, respectively, p = 0.25).

**Conclusions/Significance:**

Early detection of *Leptospira* using PCR is possible for more than half of patients presenting with leptospirosis and could contribute to individual patient care.

## Introduction

Leptospirosis is an acute febrile illness caused by pathogenic species belonging to the genus *Leptospira* [1]. This zoonotic disease has a worldwide distribution but is most common in tropical and subtropical regions and has the greatest impact on public health in developing countries [1]–[3]. Disease is maintained by chronic carrier hosts that excrete the organism into the environment, and infection in man results from direct contact with infected animals or indirect contact with a contaminated environment [1]–[3].

The accuracy of a clinical diagnosis of leptospirosis is poor because clinical features are similar to those of a range of other common infectious diseases, which in the tropical setting includes rickettsial infection, dengue and malaria. This inaccuracy has been defined in our setting in Thailand by a hospital-based study in which the clinical diagnosis of leptospirosis was correct in only 143/700 (20%) of suspected cases [4]. Several long-established diagnostic methods are available including culture of *Leptospira* spp. from blood [5], and serological testing of paired serum samples [6]. Both provide retrospective diagnostic confirmation and so do not contribute to the immediate management pathway, and culture and the gold standard serological test (microscopic agglutination test, MAT) require considerable expertise that places it within the domain of the specialist reference center.

The need for rapid diagnostics at the time of admission for patients with suspected leptospirosis has led over the last two decades to the development of numerous assays to detect antigen in a range of samples using the polymerase chain reaction (PCR). Conventional and real-time PCR have been described for the detection of *Leptospira* in blood taken from humans within the first week of clinical symptoms (when patients are leptospiremic) [7]–[18]. This reduces time to diagnosis and can be performed outside of the reference laboratory. Assays fall into two categories based on the detection of genes that are universally present in bacteria (for example, *gyrB* [16], *rrs* (16S rRNA gene) [10], [11], [15], [17] and *secY* [7]), or detection of genes that are restricted to pathogenic *Leptospira* spp. (e.g. *lipL32* [9], [18], *ligA* [13], and *ligB* [13]). Here, we compare the diagnostic sensitivity and specificity of two published real-time PCR assays targeting *rrs* [15] or *lipL32* [18] for the diagnosis of leptospirosis in Thailand. In addition, we provide insights into human disease in our population by defining the *Leptospira* spp. count in blood in relation to duration of symptoms and patient outcome.

## Results

### Analytical sensitivity and specificity

Analytical sensitivity and specificity were re-evaluated for these previously described assays because of the modifications made to the published methodology. Analytical sensitivity (limit of detection (LOD)) was reported previously as 5–20GE/reaction [15], [18]. Positive control samples were evaluated in duplicate on 13 independent occasions using DNA of *L. interrogans* serovar Lai strain Lai. For the *rrs* assay, 12/13 runs were positive for 1 GE/reaction (both samples were positive in 7/13 runs and one of two samples were positive in 5/13 runs). For the *lipL*32 assay, 12/13 runs were also positive for 1 GE/reaction (both samples were positive in 3/13 runs and one or two samples were positive in 9/13 runs). One of 13 runs was negative at the level of 1 GE/reaction for both assays. All 13 runs were positive in duplicate for 10GE/reaction for both assays. These data indicate that using duplicate samples, PCR using either assay has a sensitivity of 92% (95%CI: 64–99%) to detect 1 GE/reaction. An LOD value of 1 GE/reaction equates to 40 *Leptospira* cells per 1 ml of human blood using the DNA extraction and PCR protocol described in materials and methods. For the *rrs* assay, PCR amplification efficiency was 0.87 (slope = −3.69 [95%CI = −3.81 to −3.58], *y* intercepts = 40.24 [95%C = 39.93 to 40.54], and r-squared value = 0.98). For the *lipL32* assay, PCR amplification efficiency was 0.91 (slope = −3.57 [95%CI = 3.68 to 3.48], *y* intercepts = 40.57 [95%CI = 40.30 to 40.84], and r-squared value  = 0.99).

Analytical specificity was determined using DNA from 16 *Leptospira* strains belonging to pathogenic, intermediate or non-pathogenic *Leptospira* spp. (Table 1), together with 9 other bacterial species that frequently cause febrile illness in our population. Both assays gave a positive reaction for all pathogenic *Leptospira* spp., with a median quantification cycle (C_q_) of 16.43 (IQR 16.30–16.77) and 15.62 (IQR = 15.18–16.34) for the *rrs* and *lipL32* assay, respectively. As expected [18], intermediate *Leptospira* gave a negative reaction for the *lipL32* assay but a positive reaction for the *rrs* assay. The *lipL32* assay was negative for the non-pathogenic *Leptospira* spp. tested, but the *rrs* assay was positive for two strains (*L. terpstrae* serovar Hualin strain LT11-33 and *L. yanagawae* serovar Saopaulo strain Sao Paulo). However, the *rrs* assay C_q_ values for non-pathogenic species (median 38.00, IQR 37.50–39.64) were clearly distinct from that for intermediate group species (median 16.15, IQR 15.97–16.33). All reactions were negative for both assays using DNA from one representative each of *Staphylococcus aureus, Enterococcus* sp., *Escherichia coli, Salmonella enterica* serovar Typhi, *Klebsiella pneumoniae, Pseudomonas aeruginosa, Burkholderia pseudomallei, Orientia tsutsugamushi* strain Kato and *Rickettsia typhi*.

**Table 1:** *Leptospira* spp. used during this study.

Serovar	Serogroup	Strain	Species	Status	*rrs*	*lipL32*	Source
Lai	Icterohaemorrhagiae	Lai	*L. interrogans*	Pathogenic	+	+	Australia@
Autumnalis	Autumnalis	Akiyami A	*L. interrogans*	Pathogenic	+	+	Australia
Cynopteri	Cynopteri	3522C	*L. kirschneri*	Pathogenic	+	+	NIH*
Fortbragg	Autumnalis	Fort Bragg	*L. noguchii*	Pathogenic	+	+	Australia
Manhao3	Manhao	L60	*L. alexanderi*	Pathogenic	+	+	ATCC#
Sarmin	Sarmin	Sarmin	*L. weilii*	Pathogenic	+	+	NIH
Javanica	Javanica	Veldrat Batavia 46	*L. borgpetersenii*	Pathogenic	+	+	ATCC
Alice	Autumnalis	Alice	*L. santarosai*	Pathogenic	+	+	Australia
Pingchang	Ranarum	80–412	*L. alstonii*	Pathogenic	+	+	KIT$
Lyme	Lyme	10	*L. inadai*	Intermediate	+	−	ATCC
Korat		Khorat-H2^T^	*L. wolffii*	Intermediate	+	−	Mahidol^a^
Patoc	Semaranga	Patoc I	*L. biflexa*	Non-pathogenic	−	−	NIH
Semaranga	Samaranga	Veldrat Semarang173	*L. meyeri*	Non-pathogenic	−	−	KIT
Codice	Codice	CDC	*L. wolbachii*	Non-pathogenic	−	−	Australia
Hualin	Icterohaemorrhagiae	LT11-33	*L. terpstrae*	Non-pathogenic	(+)	−	ATCC
Saopaulo	Semaranga	Sao Paulo	*L. yanagawa*	Non-pathogenic	(+)	−	Australia

@ WHO/FOA/OIE/ Collaborating Center for Reference and Research on Leptospirosis, Australia;

*Bureau of Emerging Infection Disease, Ministry of Public Health, Thailand;

# American Type Culture Collection, USA;

$ Royal Tropical Institute (KIT), Netherland;

a Dr Thareerat Kalambaheti, Mahidol University, Thailand.

+ and − indicate a positive or negative result in the relevant PCR assay, respectively. (+) indicates a positive PCR result at a high Cq.

### Diagnostic sensitivity and specificity

**Figure 1:**
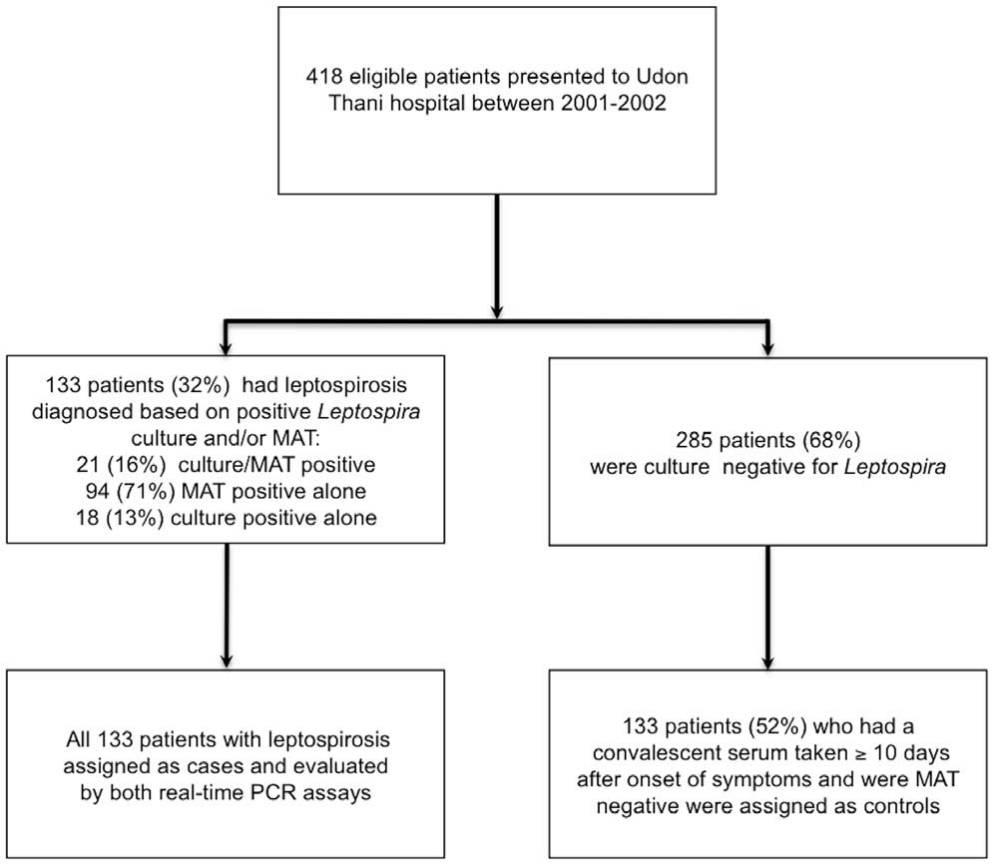
Study design and patient recruitment.

A case-control study of 266 patients (133 cases of leptospirosis and 133 controls) was constructed to evaluate the diagnostic sensitivity (DSe) and specificity (DSp) of both PCR assays for leptospirosis in Thailand (Figure 1). The median (IQR, range) age was 35 years (26–46, 15–74) for cases and 42 years (29–54, 15–79) for controls (p = 0.01). The proportion of study patients who were males was 81% and 57% for cases and controls, respectively (p<0.001). The finding that cases were predominantly male and younger than controls may relate to the fact that most cases of leptospirosis in people living in northeast Thailand occur in rice farmers and other agricultural workers. The median duration of illness prior to admission was 4 days (IQR 2–5 days; range 1–12 days) for cases and 6 days (IQR 3–9 days, range 0–33 days) for controls (p<0.001). Five cases (4%) and four controls (3%) died during hospital admission.

The DSe and DSp of the two PCR assays were determined using culture and/or MAT positivity as the gold standard (Table 2). The DSe was higher for the *rrs* assay than the *lipL32* assay (56%, (95% CI 47–64%) versus 43%, (95% CI 34–52%), p<0.001). No cases were positive for the *lipL32* assay alone. There was borderline evidence to suggest that the DSp of the *rrs* assay was lower than the *lipL32* assay (90% (95% CI 83–94%) versus 93%, (95%CI 88–97%), p = 0.06). Nine controls gave positive reactions for both assays and 5 controls gave a positive reaction for the *rrs* assay alone. Four controls positive by both assays had scrub typhus based on a four-fold rising antibody titer defined using a standard immunofluorescence assay (IFA), while the remaining 10 positive controls had fever of unknown cause.

**Table 2:** Diagnostic sensitivity and specificity of real-time PCR assays targeting *rrs* or *lipL32.*

Diagnostic test	Cases		Controls	
	Patients positive/total number tested	Sensitivity% (95%CI)	Patients positive/total number tested	Specificity% (95%CI)
*rrs* PCR assay	74/133	56 (47–64)	14/133	90 (83–94)
*lipL32* PCR assay	57/133	43 (34–52)	9/133	93 (88–97)
Culture	39/133	29 (22–38)	0/133	100
MAT	115/133	86 (79–92)	0/133	100
*rrs* PCR assay for leptospiremia	37/39	95 (83–99)	-	-
*lipL32* PCR assay for leptospiremia	34/39	87 (73–96)	-	-

Culture and PCR both detect the presence of leptospiremia and represent a more direct comparison than PCR versus serological tests on paired sera. We reanalyzed the DSe of the two PCR assays in 39 leptospiremia cases in which testing was performed on parallel samples taken on admission. The DSe of the *rrs* and *lipL32*assays were high in this subgroup and not significantly different (95% and 87%, respectively, p = 0.25). Furthermore, both PCR assays were clearly more sensitive than culture overall, with antigen detection by PCR of an additional 37 and 23 culture-negative cases for *rrs* and *lipL32*, respectively.

We considered whether PCR could have provided a rapid diagnosis of leptospirosis in the 5 patients with fatal infection. Both PCR assays were positive in 4 cases and negative for 1 case. The latter patient presented with a five-day history of fever, was culture negative for *Leptospira*, and had an admission MAT titer of 1∶400.

One possible explanation for the false negative PCR results in patients who were leptospiremic is that the infecting species in these cases were distantly related to pathogenic strains of genus *Leptospira* with failure of primer or probe binding at a region of genetic diversity. This did not appear to be the case, however, since 2 cases who were culture positive but negative by both PCR assays were infected by *L. interrogans* serovar Autumnalis that had been characterised previously using multilocus sequence typing as sequence type (ST) 34, the dominant *Leptospira* clone associated with human disease in Thailand [19]. A further 3 cases who were positive for the *rrs* assay but negative by the *lipL32* assay were also infected by *L. interrogans* serovar Autumnalis (ST 34 (n = 2) or ST 41 (n = 1)) [19].

### Quantification of Leptospira in clinical blood samples

Quantitative data are presented for the *rrs* assay. The median number of *Leptospira* in admission blood of PCR positive cases was 994.5 GE/ml (range 47–970,112; IQR 200–6,215 [n = 74]). There was borderline evidence suggesting that the copy number was higher in patients who were culture positive (median 1,136 GE/ml, IQR 404–6,656 [n = 37]) than culture negative (median 358 GE/ml, IQR = 115–2,299 [n = 37], p = 0.072).

The median number of *Leptospira* in the admission blood sample from *rrs* positive controls (median 1,153 GE/ml; range 55–8,711; IQR 94–5,558, [n = 14]) was comparable to that for *rrs* positive cases (p = 0.49).

### Window of positivity

Duration of illness prior to admission was considered in relation to a positive *rrs* assay result for 129 cases with available clinical data. Cases with a positive reaction (n = 73) reported the presence of symptoms for a median of 3 days (IQR 2–4 days) prior to hospital presentation. This was shorter than that for cases who were *rrs* negative (median 5 days, IQR 3.5–6 days [n = 56]) (p<0.001). These findings were reproduced for the *lipL32* assay (data not shown). Similarly, cases who were culture positive for *Leptospira* (n = 39) had a shorter duration of illness than cases who were culture negative (n = 90) (median 2 days, IQR = 1–3 days vs. median 4 days, IQR 3–6 days (p<0.001). Comparison of the window of positivity for culture versus PCR demonstrated that PCR outperformed culture, in that PCR positive/culture positive cases had a shorter duration of illness than PCR positive/culture negative cases (median 2 days, IQR 1–3 days vs. median 4 days, IQR 3–5 days) (p<0.001).

Controls who were positive for the *rrs* assay had a comparable duration of symptoms to PCR positive cases (median 2 days, IQR 2–4 days; n = 14) (p = 1.0).

## Discussion

Reassessment of analytical sensitivity and specificity demonstrated that the two PCR assays performed at least as well in our laboratory as in the original publications [15], [18]. The LOD was comparable between the two assays in our hands, although the initial publications reported that the LOD was lower for *rrs* than for *lipL32* (5 vs. 20 GE/reaction, respectively). *L. interrogans* has two copies of *rrs* but a single copy of *lipL32*, and this may have been responsible for the very subtle difference in the LOD of the two assays (for the *rrs* assay, 5/13 and 7/13 runs were positive for 1 GE/reaction in single and duplicate samples, respectively, compared with 3/13 and 9/13 runs for the *lipL*32 assay). An obvious difference between the two assays is that the target for the *rrs* assay is ubiquitous among *Leptospira* spp. while the *lipL32* assay would only be predicted to be positive for the pathogenic group and not the intermediate and non-pathogenic groups. We are currently defining the species of *Leptospira* causing disease in Thailand and cannot yet exclude the possibility that culture-negative patients who were positive by *rrs* but negative by *lipL32* were infected with a species belonging to the intermediate group.

Our finding that PCR had a lower diagnostic sensitivity than MAT is consistent with previous reports [7]. Plausible explanations include late presentation associated with absence of *Leptospira* in blood, and pre-treatment with antimicrobial drugs prior to admission. A wide range of oral antibiotics is available over the counter in Thailand, and self-medication prior to hospital presentation is common.

The quantification of *Leptospira* in blood during this study was a useful exercise, since this can provide critical baseline information during the development of point of care antigen detection tests. The finding that the bacterial count was higher in patients who were culture positive compared with those who were culture negative was intuitive.

Our data on the window of PCR or culture positivity after the onset of symptoms suggest that these tests only have clinical utility within the first week of clinical manifestations, as reported previously [1], [3]. We observed that the period over which PCR was positive after the start of symptoms was longer than that for culture. A small number of patients were positive by culture but negative by PCR. However, the difficulty and expense of culture combined with the prolonged delay before culture becomes positive means that culture results will not influence individual patient care.

The basis for a negative PCR result but positive culture remains unexplained, but possible explanations include a very low count in the initial sample associated with a stochastic effect in which the organism was present in the aliquot taken for culture but not for PCR. It is also possible that PCR inhibitors were present that interfered with the detection of a very low copy number but did not affect the detection of the positive control DNA (*rnaseP*), which would be present in abundance. The basis for positive PCR results in patients who were negative by culture and MAT and who had another diagnosis or unknown diagnosis is also uncertain. One possibility is that some patients had more than one infection and false negative diagnostic tests for leptospirosis. It is quite possible that patients could develop both leptospirosis and scrub typhus in the same timeframe since agricultural workers are often exposed to the pathogens causing both infections. Previous studies have documented patients with serological evidence for concurrent leptospirosis and scrub typhus [20], but the putative situation in which patients have more than one infection but negative diagnostic tests for leptospirosis is speculative and extremely difficult to prove. An alternative explanation is laboratory contamination, although the negative controls remained negative throughout the study and the stage of any contamination event would have to have been at an earlier part of the study pathway (for example, during DNA extraction).

Making an accurate diagnosis of leptospirosis contributes to both the characterization of disease epidemiology and to individual patient care. The diagnosis of leptospirosis across much of Thailand continues to be made on the basis of clinical features because of a lack of inexpensive and easy to use diagnostics tests. MAT is performed by the National Institute for Health, Thailand and is available as a reference test, but is used for a minority of suspected cases overall to underpin epidemiological data and provides a retrospective diagnosis. *Leptospira* culture is largely a research activity, and has no clinical utility in relation to immediate patient care. The PCR assays evaluated in this study confirmed the diagnosis of leptospirosis in half of definite cases, and further studies are now required to determine whether such information would have altered patient morbidity and mortality, together with the effect of false positive test results. The feasibility of introducing PCR tests, however, rests on affordability; the cost of introducing a test into laboratories that do not currently perform PCR would be high both in terms of equipment and training.

In conclusion, *Leptospira* detection using PCR could improve the management of patients presenting to hospital within the first few days of the onset of symptoms of leptospirosis, although cost represents a barrier to its implementation in resource-restricted countries. An on-going study is currently evaluating the diagnostic sensitivity and specificity of LAMP (loop-mediated isothermal amplification), a technique that requires minimal equipment and modest training.

## Methods

The Standards for the Reporting of Diagnostic accuracy testing (STARD) were followed during the conduct of this study [21].

### Laboratory strains and DNA extraction

The *Leptospira* spp. used during this study are listed in Table 1. Additional isolates used were one clinical isolate of each of the following bacterial species: *S. aureus, Enterococcus* sp., *E. coli, S. enterica* serovar Typhi, *K. pneumoniae, P. aeruginosa, B. pseudomallei, O. tsutsugamushi* strain Kato and *R. typhi*. Genomic DNA was extracted from laboratory cultures using the Wizard® Genomic DNA extraction kit (Promega, USA), with the addition of 5 µl of 10mg/ml lysostaphin during the extraction of *S. aureus*.

### Clinical evaluation of PCR assays

Patients with laboratory confirmed leptospirosis (cases) or without leptospirosis (controls) were drawn from a prospective cohort study of 418 consecutive patients presenting to the Udon Thani hospital, northeast Thailand with an acute febrile illness between 10^th^ January 2001 and 16^th^ June 2002 [19]. In brief, patients were recruited into the study during twice daily ward rounds. Inclusion criteria were patients who were ≥15 years of age with fever (>37.8°C) of unknown cause who agreed to participate and to attend out-patient follow up for a convalescent serum sample. Exclusion criteria were patients with a blood smear positive for malaria parasites or those with another definable source of infection on admission such as pneumonia or urinary tract infection.

Parallel blood samples for culture, MAT and PCR assays were taken on admission from all patients for *Leptospira* culture, serological testing and molecular diagnostics, and a second (convalescent) sample was taken for serological testing around 2 weeks later. *Leptospira* culture was performed using a heparin blood sample taken on admission as described previously [22], and isolates sent to the WHO/FAO/OIE Collaborating Center for Reference & Research on Leptospirosis, Australia for serovar identification using the cross agglutinin absorption test (CAAT).^6^ The microscopic agglutination test (MAT) was performed by the WHO/FAO/OIE Collaborating Center for Reference & Research on Leptospirosis, Australia, as described previously [6], using a live panel of antigens representing both ubiquitous and locally prevalent serovars. A diagnosis of leptospirosis was based on isolation of *Leptospira* from blood and/or a positive MAT, which was defined as a 4-fold rise in titer between acute and convalescence samples or a single titer of ≥1∶400. Patients who did not meet these criteria were defined as not having current or recent leptospirosis.

All patients enrolled into the prospective cohort study who were diagnosed as having leptospirosis (n = 133) were selected as cases. Laboratory confirmation was made on the basis of being positive by culture and MAT in 21 (16%) patients, culture positive/MAT negative in 18 (13%) patients, and culture negative/MAT positive in 94 (71%) patients. A positive MAT result was based on a 4-fold rising titer in 97 cases and a single titer of ≥1∶400 in 27 cases. Controls (n = 133) were randomly selected from those patients who did not meet the diagnostic criteria for leptospirosis. All patients in this group had a convalescent serum sample taken a median of 17 days (range 10–43, IQR = 13–21) after the onset of symptoms. The discharge diagnoses of controls were as follows: scrub typhus (n = 54), bacterial septicemia (n = 8) (*Escherichia coli* (n = 2), *Klebsiella pneumoniae* (n = 2), *Acinetobacter baumanii* (n = 1), *Corynebacterium jeikeium* (n = 1), *Enterococcus* sp. (n = 1), or *Streptococcus pneumoniae* (n = 1)), dengue fever (n = 5), murine typhus (n = 4), melioidosis (n = 2), HIV-related infections (n = 2), other diagnoses (n = 7), and unknown diagnosis (n = 51). A database was created in which cases and controls were entered, randomized and blinded to the technician prior to performing the two real-time PCR assays.

Whole blood samples collected from cases and controls on admission were drawn into a 5.0 ml tripotassium EDTA 15×54 mm tube (Teklab, UK). Samples were left at room temperature for no more than 6 hours prior to storage at −80°C. The sample was thawed and DNA extracted in 2009. Extraction was performed using the Nucleon™ BACC Genomic DNA Extraction Kit (GE Healthcare Biosciences, USA), and the extract suspended in 1 ml of TE buffer. DNA samples were stored at -20°C prior to PCR assays. The technician who performed DNA purification were blinded to case-control grouping data. The efficiency of DNA extraction from patient blood samples was evaluated using a real-time PCR assay targeting the homo sapiens ribonuclease P (*rnaseP*) gene, as described previously [18]. This was positive for all 266 clinical samples.

### PCR assays

Molecular assays were performed at the Mahidol-Oxford Tropical Medicine Research Unit. Two previously published real-time PCR assays that used hydrolysis probes (TaqMan probes) targeting either *rrs* or *lipL32* were evaluated [15], [18]. Both assays were performed using the primers and probe described previously [15], [18]. Assay modifications were as follows: Platinum *Taq* DNA Polymerase (Invitrogen, Brazil) was used as the master mix, and for the *rrs* assay the Mg concentration was reduced from 4.50 to 4.25 mM, the forward primer reduced from 1 µM to 0.25 µM, the reverse primer reduced from 1 µM to 0.50 µM and the probe reduced from 0.20 µM to 0.05 µM. A 20 µl reaction mixture contained 5 µl of DNA extracted from laboratory cultures or EDTA blood samples taken from febrile patients. Both assays were performed using the Rotor-GeneTM3000 Real Time Thermal Cycler (Corbett Life Science, Australia) using the following cycling conditions: 98°C for 1 minute, followed by 45 cycles of 95°C for 15 seconds and 58°C for 1 minute, then 25°C for 20 seconds. The intensity of 6-carboxy-fluorescein (FAM) was acquired at the end of each 58°C step. Genomic DNA extracted from *L. interrogans* serovar Lai strain Lai was used as a positive control and quantification calibrator for the estimation of bacterial copy number in clinical samples. Positive control DNA was quantified using spectrophotometry (NanoDrop; Thermo Scientific). Ten-fold serial dilutions of the DNA were prepared using healthy human DNA as diluent; blood was taken from a single individual and extracted as described above for clinical samples. The amount of calibrator (GE/µl) was estimated based on a genomic size of 4.659 Mb for *L. interrogans* serovar Lai strain Lai [23]. A calibration curve was constructed by plotting the logarithmic value of bacterial copies versus C_q_. This revealed a linear assay over 5 orders of magnitude (1 to 1×10^4^ GE/reaction). PCR amplification efficiency was established by means of calibration curves [24]. Reaction mixture minus DNA template was used as a negative control. All patient samples, positive and negative control reactions were performed in duplicate for both PCR assays by a single technician who was blinded to case-control grouping data. A positive PCR result was defined when one or both duplicates had a FAM signal above a fixed threshold of 0.1.

### Ethics Statement

Ethical approval for the cohort study was obtained from the Ministry of Public Health, Royal Government of Thailand, and the Oxford Tropical Research Ethics Committee, UK. Written inform consent was obtained from each subject enrolled into the study [19].

### Statistical analysis

Statistical analyses were performed using STATA/SE version 10.0 (College Station, Texas, United States). Diagnostic sensitivity and specificity of each PCR assay was defined against the combined result for culture and MAT (a positive result for either or both being interpreted as diagnostic for leptospirosis), and expressed as a proportion with exact 95% confidence intervals (CI). Comparison of diagnostic sensitivity and specificity between the two PCR assays was performed using the McNemar test. Comparison of the duration of illness prior to hospital presentation between patients with laboratory-confirmed leptospirosis who were positive by PCR and/or culture was performed using the Mann-Whitney test.
