# Correction: Diagnostic Accuracy of Real-Time PCR Assays Targeting 16S rRNA and *lipl32* Genes for Human Leptospirosis in Thailand: A Case-Control Study

**DOI:** 10.1371/annotation/e2b77844-576a-4d20-b698-09ad057872fc

**Published:** 2011-02-16

**Authors:** Janjira Thaipadungpanit, Wirongrong Chierakul, Vanaporn Wuthiekanun, Direk Limmathurotsakul, Premjit Amornchai, Siriphan Boonslip, Lee D. Smythe, Roongrueng Limpaiboon, Alex R. Hoffmaster, Nicholas P. J. Day, Sharon J. Peacock

The first author's name is misspelled. The correct spelling is: Janjira Thaipadungpanit. The correct citation is: Thaipadungpanit J, Chierakul W, Wuthiekanun V, Limmathurotsakul D, Amornchai P, et al. (2011) Diagnostic Accuracy of Real-Time PCR Assays Targeting 16S rRNA and lipl32 Genes for Human Leptospirosis in Thailand: A Case-Control Study. PLoS ONE 6(1): e16236. doi:10.1371/journal.pone.0016236

